# The association between adiponectin, HDL-cholesterol and α1-antitrypsin-LDL in female subjects without metabolic syndrome

**DOI:** 10.1186/1476-511X-9-147

**Published:** 2010-12-30

**Authors:** Kazuhiko Kotani, Toshiyuki Yamada, Nobuyuki Taniguchi

**Affiliations:** 1 Department of Clinical Laboratory Medicine, Jichi Medical University, Tochigi, Japan

## Abstract

**Background:**

Oxidized low-density lipoprotein (LDL) may act as an atheroprotective (anti-atherosclerotic) agent under some conditions. While the α1-antitrypsin (AT)-LDL complex is considered a type of oxidized LDL, its clinical relevance remains unknown. The aim of the present study was to investigate the association between AT-LDL and anti-atherosclerotic variables such as HDL-cholesterol and adiponectin in subjects with and without metabolic syndrome (MetS).

**Methods:**

In asymptomatic females (n = 194; mean age, 54 years) who were divided into non-MetS (n = 108) and MetS groups (n = 86), the fasting levels of serum AT-LDL, adiponectin and glucose/lipid panels were measured, in addition to body mass index (BMI) and blood pressure.

**Results:**

The MetS group showed significantly higher BMI, blood pressure, glucose and triglyceride levels as well as significantly lower levels of HDL-cholesterol and adiponectin than the non-MetS group. A multivariate-adjusted analysis revealed that in the non-MetS group, AT-LDL was significantly, independently and positively correlated with adiponectin (β = 0.297, P < 0.05), along with HDL-cholesterol (β = 0.217, P < 0.05). In the MetS group, AT-LDL was significantly, independently and positively correlated with LDL-cholesterol only (β = 0.342, P < 0.05).

**Conclusions:**

These data suggest that AT-LDL may exert anti-atherosclerotic effects in female subjects without MetS. More studies are required to clarify the clinical roles of AT-LDL in relation to the pathophysiology of MetS.

## Background

Atherosclerosis is an important health concern, because it leads to cardiovascular disease [1]. Oxidation of low-density lipoprotein (LDL) is involved in the atherosclerotic processes, and oxidized LDL (oxLDL) can exhibit atherogenic properties [2,3]. Recent research has shown that various types of oxLDL exist both in atherosclerotic lesions and in the circulation, and the circulating oxLDL is presently thought to be a useful marker reflecting one's atherosclerotic state [4-6]. On the other hand, it is becoming increasingly clear that oxLDL exerts dual effects on atherosclerosis [7-15]. Namely, low levels (small amounts) of oxLDL can exert an atheroprotective (anti-atherosclerotic) effect. However, the clinical data about this phenomenon are limited, and more clinical investigations are needed.

While α1-antitrypsin (AT), a serine-proteinase inhibitor, protects tissues from damage caused by excess activities of proteinases [16], when AT is oxidized, it loses its inhibitory activity and activates monocytes [17]. Oxidized AT has been shown to form a complex with LDL in atherosclerotic lesions, and the circulating AT-LDL complex seems to reflect the atherosclerotic state, although the precise mechanisms underlying the formation of the AT-LDL complex remain undetermined [18]. Importantly, the clinical significance of AT-LDL, as an oxLDL marker, in atherosclerosis is unknown. In our previous study of a specific atherosclerosis-prone population with obesity and metabolic syndrome (MetS), we did not find any significant role for AT-LDL [19]. However, in agreement with the original report that developed the AT-LDL measurement system [18], we have also noted that the circulating levels of AT-LDL were very low in these patients [19].

This prompted us to investigate the association between circulating AT-LDL and anti-atherosclerotic variables, such as high-density lipoprotein (HDL) and adiponectin [20]. The levels of these variables generally differ between subjects with and without MetS (in particular, adiponectin has recently attracted much attention because of its association with MetS) [20]. The clinical relationship between AT-LDL and these variables should be examined with regard to a potential role in MetS. Therefore, the aim of the present study was to determine whether there was a correlation between AT-LDL and atherosclerotic variables, including HDL-cholesterol and adiponectin, in subjects with and without MetS.

### Subjects and Methods

A total of 194 non-medicated and asymptomatic female participants, ranging from 35-70 years of age, were recruited during routine check-ups in the health education classes and outpatient clinics. The study population was composed of subjects without MetS (n = 108) and with MetS (n = 86) (Table 1). The eligible subjects had no histories of cardiovascular, thyroid, hematological, kidney or liver diseases. The existence of 3 out of 5 of the following criteria constituted a diagnosis of MetS according to the National Cholesterol Education Program Adult Treatment Panel (NCEP-ATP) III recommendations [21]: 1) obesity identified by a body mass index (BMI) ≥ 25.0 kg/m^2 ^as a surrogate for Japanese subjects [22], 2) elevated blood pressure (BP) identified by systolic BP (SBP) ≥ 130 mmHg and/or diastolic BP (DBP) ≥ 85 mmHg, 3) hypertriglyceridemia identified by serum triglyceride (TG) ≥ 1.69 mmol/L, 4) decreased HDL-cholesterol identified by serum HDL-cholesterol <1.29 mmol/L, and 5) elevated glucose status identified by a fasting plasma glucose (FPG) ≥ 6.1 mmol/L. The study was approved by the Jichi Medical University ethics committee, and all subjects gave their informed consent.

**Table 1:** Clinical characteristics in subjects with and without MetS

Variable	Non-MetS (n = 108)	MetS (n = 86)	P value
Age, years	54.1 ± 9.6	53.4 ± 9.5	0.618
Current smoking, %	9.3	10.5	0.779
Body mass index, kg/m^2^	26.0 ± 2.5	27.5 ± 1.7	<0.0001**
Systolic blood pressure, mmHg	130 ± 17	146 ± 17	<0.0001**
Diastolic blood pressure, mmHg	79 ± 11	87 ± 10	<0.0001**
Fasting plasma glucose, mmol/L	5.85 ± 2.30	7.42 ± 3.10	<0.0001**
LDL-cholesterol, mmol/L	3.32 ± 0.80	3.38 ± 0.77	0.628
Triglyceride, mmol/L	1.15 (0.88-1.47)	1.88 (1.36-2.47)	<0.0001**
HDL-cholesterol, mmol/L	1.71 ± 0.33	1.48 ± 0.38	<0.0001**
Adiponectin, μg/mL	8.95 (5.53-12.2)	7.80 (4.80-10.9)	0.050*
AT-LDL, μg/mL	3.27 ± 1.02	3.00 ± 0.98	0.071

MetS: metabolic syndrome, LDL: low-density lipoprotein, HDL: high-density lipoprotein, α1-AT-LDL: α1-antitrypsin-low-density lipoprotein. Data are expressed as the mean ± standard deviation, the median (interquartile range) or the percentage in each subject group. Significance level (subjects without MetS *vs*. those with MetS; unpaired t-test [triglyceride and adiponectin were log-transformed] or χ^2 ^test): * P ≤ 0.05, ** P ≤ 0.01.

Current smoking was defined as the presence of current smoking habits via a self-report and professional interview. In addition to the BMI, the SBP and DBP levels were determined in the seated subject's right-arm with a mercury sphygmomanometer after 5 minutes of rest. During an overnight fast, the serum TG and FPG levels were measured using enzymatic methods, and the serum LDL-cholesterol and HDL-cholesterol levels were measured using homogeneous methods. The serum adiponectin levels were measured with an enzyme-linked immunosorbent assay (Otsuka Pharmaceutical Co. Ltd., Tokyo, Japan). Serum AT-LDL levels were determined according to an enzyme-linked immunosorbent assay method, as described previously [18,19]. Briefly, fresh serum samples (50 L/well) were incubated overnight at 4°C with the anti-human AT-specific antibody (DAKO Denmark A/S, Glostrup, Denmark: AT-LDL, clone No.27) coated onto a microtiter plate. After washing, a biotinylated Fab' anti-human apo-B antibody was added as the capture antibody (100 L/well), and reacted for 2 hours at room temperature. The activity was measured in terms of the difference in optical absorbance between 450 and 620 nm after adding a peroxidase substrate, when the reaction was terminated by adding phosphoric acid. The intraassay coefficients of variation at low and high concentrations of AT-LDL were 1.8% and 1.6%, respectively. The interassay coefficients of variation at low and high concentrations of AT-LDL were 5.9% and 5.4%, respectively. All samples were assayed in duplicate and in random order.

The data were expressed as the means ± standard deviation (SD) or the median plus interquartile range. The data between the subject groups were compared using an unpaired t-test. A simple and multivariate-adjusted linear regression model was utilized to observe the correlation between AT-LDL and other atherosclerotic variables. All of the measured variables, except for DBP (because of its close collinearity to SBP: correlation coefficient >0.6), were entered into the multivariate-adjusted analysis model. The levels of TG and adiponectin were log-transformed for all of the analyses because of their skewed distributions. A P value ≤0.05 was considered to be statistically significant.

## Results

With regard to the clinical characteristics of the subject groups with and without MetS (Table 1), subjects with MetS had significantly higher BMI, SBP, DBP, FPG and TG levels than those without MetS. The subjects with MetS showed significantly lower levels of HDL-cholesterol and adiponectin than those without MetS. There were no significant differences in AT-LDL levels between the two subject groups.

In the simple linear regression analysis (Table 2), AT-LDL was significantly and positively correlated with LDL-cholesterol, HDL-cholesterol and adiponectin in the subjects without MetS. In the group with MetS, AT-LDL was significantly and positively correlated with LDL-cholesterol only. In a subsequent multivariate-adjusted analysis (Table 2) in the subject group without MetS, the AT-LDL was significantly, independently and positively correlated with adiponectin, as well as HDL-cholesterol. In the group with MetS, AT-LDL remained significantly, independently and positively correlated with LDL-cholesterol only.

**Table 2:** Correlations of AT-LDL with other atherosclerotic variables

	Non-MetS		MetS	
	
Variable	γ (P value)	β (P value)	γ (P value)	β (P value)
Age, years	-0.025 (0.8001)	-0.127 (0.229)	-0.125 (0.251)	-0.197 (0.086)
Current smoking, %	-0.008 (0.934)	0.033 (0.734)	0.101 (0.356)	0.116 (0.287)
Body mass index, kg/m^2^	-0.073 (0.452)	0.081 (0.447)	-0.161 (0.138)	-0.104 (0.346)
Systolic blood pressure, mmHg	0.104 (0.285)	0.008 (0.950)	0.017 (0.875)	0.104 (0.337)
Diastolic blood pressure, mmHg	0.107 (0.269)	-	-0.131 (0.229)	-
Fasting plasma glucose, mmol/L	-0.017 (0.863)	0.081 (0.413)	-0.040 (0.771)	0.080 (0.464)
LDL-cholesterol, mmol/L	0.206 (0.033)*	0.144 (0.128)	0.349 (0.001)**	0.342 (0.002)**
Triglyceride, mmol/L	-0.022 (0.819)	0.016 (0.860)	0.204 (0.061)	0.167 (0.120)
HDL-cholesterol, mmol/L	0.303 (0.001)**	0.217 (0.048)*	-0.030 (0.784)	-0.006 (0.965)
Adiponectin, μg/mL	0.330 (<0.0001)**	0.297 (0.010)**	-0.041 (0.706)	-0.045 (0.693)

AT-LDL: α1-antitrypsin-low-density lipoprotein, MetS: metabolic syndrome, LDL: low-density lipoprotein, HDL: high-density lipoprotein. r: simple linear regression coefficient between α1-AT-LDL and another variable, β: multiple linear regression coefficient in a multivariate analysis for α1-AT-LDL after controlling for all the listed variables except for diastolic blood pressure. Triglyceride and adiponectin were log-transformed for analyses. Significance level: * P ≤ 0.05, ** P ≤ 0.01.

## Discussion

The present study is the first demonstration that AT-LDL had a significantly positive correlation with HDL-cholesterol and adiponectin in subjects without MetS, but not in those with MetS. In this study, subjects with MetS were more predisposed to be atherosclerotic, based on their BMI, BP, glucose/lipid panels and adiponectin, compared to those without MetS, which is in agreement with previous reports [20]. In addition, the finding of a significant correlation between AT-LDL and LDL-cholesterol in subjects with MetS was similarly observed in a previous study [19].

The present study provided two new findings. The first is the atheroprotective relevance of oxLDL. Earlier studies reported a significant inverse correlation between adiponectin and oxLDL in patients with type 2 diabetes mellitus and coronary artery disease [23] and in patients with chronic heart failure [24]. The significant inverse correlation between HDL-cholesterol and oxLDL was also reported in patients with coronary artery disease [25]. Our present results showing the significant positive correlation between adiponectin or HDL-C and AT-LDL would not have been expected from these reports [23-25], although the examined populations and oxLDL markers were different between the prior work and our current study. However, since relatively low levels of oxLDL can be atheroprotective [7-15], it is important to note the possibility that AT-LDL may exert anti-atherosclerotic effects in subjects without MetS. This might be an important case showing an atheroprotective aspect of oxLDL in the clinical setting.

Based on the current study alone, we cannot completely determine whether the significant relationship between adiponectin, HDL-cholestreol and AT-LDL is a simple (non-causative) correlation or a positive pathological link. However, we have considered the possible biological mechanisms of such a relationship. Oxidized LDL, as anti-atherosclerotic agent, acts through its cytoprotection, the modulation of immunological system and the activation of reverse cholesterol transport, as was well-documented in Yu's study [13]. The cytoprotective mechanism of oxLDL involves the regulation by the electrophile response element, including heme oxygenase-1 [9,26]. The atheroprotective effects of the immunity to oxLDL are known to be induced, for instance, by the secretion of anti-inflammatory cytokines, including interleukin-10 [11,13]. There is a growing appreciation that heme oxygenase-1 plays a crucial anti-inflammatory role in chronic inflammatory pathologies induced by various factors (i.e., oxidative stress and cytokines) and is a mediator of interleukin-10 expression [27,28]. While adiponectin induction by oxLDL remains to be established, the key molecules (such as heme oxygenase-1) can show a positive association with adiponectin [29]. Thus, the adiponectin/interleukin-10/heme oxygenase-1 pathway may, at least in part, explain the positive relationship between adiponectin and oxLDL. Furthermore, a report demonstrating that low levels of oxLDL increased cholesterol efflux in adipocytes has suggested that oxLDL has an atheroprotective role by enhancing the reverse cholesterol transport, thereby increasing HDL-cholesterol [13,14]. This may partly account for the positive relationship between HDL-cholesterol and oxLDL.

The second major finding of our present study is the differences in associations of oxLDL with anti-atherosclerotic variables in subjects with and without MetS. The present results showing a diminished correlation between adiponectin or HDL-cholesterol and AT-LDL in subjects with MetS are interesting, and suggest that the metabolic dysregulation and homeostatic shifts specifically found in MetS subjects (i.e., lower adiponectin or HDL-cholesterol concentrations [20]) may eliminate the significant link between them found in those without MetS. Whether the pathophysiology of MetS could specifically affect the correlation pattern of atherosclerotic variables to AT-LDL remains to be explored.

The present study had a few limitations. The cross-sectional design did not allow for a strict cause-and-effect conclusion to be drawn. The study was conducted in female subjects, and we did not obtain any data on males. There are also ethnic differences in some influences of atherosclerotic variables, including adiponectin and cholesterol, on atherosclerotic states [30]. Therefore, more studies with various populations and prospective designs are called for when carrying out future studies.

## Conclusions

In summary, the present study showed that AT-LDL was significantly, independently and positively correlated with adiponectin and HDL-cholesterol in female subjects without MetS, but not in those with MetS. These data suggest that AT-LDL may exert anti-atherosclerotic effects in subjects without MetS. Further research is warranted to confirm these findings and their relevance in the clinical and basic settings.

## Competing interests

The authors declare that they have no competing interests.

## Authors' contributions

All authors contributed to the intellectual development of this work, and approved the final manuscript. KK, TY and TN analyzed the data. KK searched the literature and wrote the draft paper. TY and TY provided critical corrections to the manuscript.
